# A Multi-Center Retrospective Observational Analysis of Three-Year Experience of Our Protocol for Prevention and Monitoring of Surgical Site Infections in Implant-Based Breast Reconstruction

**DOI:** 10.3390/cancers16132439

**Published:** 2024-07-02

**Authors:** Stefano Bottosso, Giulia Benedetta Sidoti, Ludovica Vita, Alessandro Scian, Luigi Bonat Guarini, Nadia Renzi, Vittorio Ramella, Giovanni Papa

**Affiliations:** 1 Plastic Surgery Department, Ospedale di Cattinara, ASUGI, Strada di Fiume, 447, 34149 Trieste, Italy; 2 Department of Medical, Surgical and Health Sciences, Plastic and Reconstructive Surgery Unit, University of Trieste, 34149 Trieste, Italy

**Keywords:** prevention, infection, protocol, breast surgery, reconstruction

## Abstract

**Simple Summary:**

With the increase in the mastectomy rate, the number of patients opting for postmastectomy reconstruction has been rising, with implant-based procedures being the most commonly used method. One of the most feared complications is the loss of reconstruction, often caused by implant infection. This can lead to prolonged antibiotic treatments, additional surgical procedures, capsular contracture, and unsatisfactory aesthetic results, with a significant psychological impact on patients. This study analyzed infection rates at our institution and evaluated the effectiveness of our prevention protocol introduced in 2020, comparing the data on surgical site infections (SSIs) between Trieste Hospital (with the protocol) and another center in Gorizia (without the protocol). We enrolled 396 patients, divided into the experimental group (Trieste) and the control group (Gorizia). Infected patients were 5 in the first group (1.7%) and 8 in the second group (7.9%), with an overall infection rate of 3.2%. We conclude that the prevention protocol has reduced the incidence of postoperative infections.

**Abstract:**

Background: With the rise in the mastectomy rate, the number of patients who choose to undergo postmastectomy reconstruction has been increasing, and implant-based procedures are the most performed methods for postmastectomy breast reconstruction. Among the possible complications, the most feared is the loss of reconstruction. It can be related to several reasons, but one of the most common is infection of the implant, which can lead to prolonged antibiotic treatment, undesired additional surgical procedures, increased incidence of capsular contracture, and unsatisfactory aesthetics results, with a huge psychological impact on patients. Aims: The primary intent of this study is to analyze the status of infection rates at our institution and evaluate the effectiveness of our prevention protocol since its introduction. Secondly, we compared data of the surgical site infections (SSIs) after implant-based breast reconstruction at Trieste Hospital, where the protocol has been employed since 2020, and in another center, where plastic surgeons of our team are involved, with different prevention procedures. Methods and Results: We enrolled 396 female patients, who underwent implant-based breast reconstruction, using definitive mammary implants or breast tissue expanders, with or without ADM (acellular dermal matrix), both for breast cancer and risk-reducing surgery in BRCA1/2 patients. Patients treated at the Hospital of Trieste, with the use of the prevention protocol, were considered the experimental group (group 1), while patients treated in Gorizia by the same breast team with standardized best-practice rules, but without the use of the prevention protocol, were considered the control group (group 2). Infected patients were 5 in the first group (1.7%) and 8 in the second one (7.9%), with a global infection rate of 3.2%. Conclusion: After the introduction of our prevention protocol, we faced a lower incidence of infection after breast surgery with implants or tissue expanders.

## 1. Introduction

In recent decades, breast surgery for oncologic or prophylactic purposes has significantly increased. With the rise in mastectomy rates, more patients are opting for postmastectomy reconstruction [1], predominantly utilizing implant-based procedures. One of the most serious complications is the failure of reconstruction, often due to implant infection. This can lead to prolonged antibiotic treatment, additional surgeries, increased capsular contracture, unsatisfactory aesthetic outcomes, and a substantial psychological impact on patients [2,3]. Reported infection rates for breast prosthetics range from 1% to 35% [3,4,5]. Approximately 50% of explantations occur within 30 days of surgery, with 9% occurring more than 100 days later [5]. Commonly isolated organisms from infected breast implants include *Staphylococcus* spp., notably, *S. aureus* and coagulase-negative staphylococci [6,7,8], with a rising incidence of methicillin-resistant *S. aureus* (MRSA) infections, which are associated with lower implant salvage rates [9]. Additionally, Gram-positive bacteria such as *Streptococcus* spp. and Gram-negative bacteria like *Pseudomonas* spp. are increasingly reported [6,7,8]. Numerous studies have examined patient demographics, comorbidities, and surgical procedures affecting infection risks [10,11,12]. Our previous study described a prevention protocol based on patient risk factors with promising results [13].

This study aims to analyze the infection rate status at our institution and evaluate the effectiveness of our prevention protocol since its introduction. Additionally, we compared SSI data after implant-based breast reconstruction at Trieste Hospital, employing the protocol since 2020, with another center where different prevention procedures are utilized.

## 2. Materials and Methods

Over a three-year period, from June 2020 to July 2023, we conducted a multi-centric retrospective observational study at the Plastic and Reconstructive Surgery Unit of Azienda Sanitaria Universitaria Giuliano Isontina (ASUGI) in Trieste (Italy) and at Gorizia Hospital (Italy).

The study was in full accordance with the Helsinki declaration and was approved by the Health Department and the administrative direction of the Cattinara Hospital of Trieste.

We enrolled 396 female patients, who underwent implant-based breast reconstruction, using definitive mammary implants or breast tissue expanders, with or without ADM (acellular dermal matrix), both for breast cancer and risk-reducing surgery in BRCA1/2 patients.

The exclusion criteria were missing data, inflammatory breast cancer, autologous reconstructions, and mastectomy without any reconstruction.

Patients treated at the Hospital of Trieste, with the use of the prevention protocol, where considered the experimental group (group 1), while patients treated in Gorizia by the same breast team with standardized best-practice rules, but without the use of the prevention protocol, were considered the control group (group 2). Informed consent was obtained from all subjects involved in the study.

The prevention protocol for SSIs for group 1 patients involves a pre-operative phase concerning MSSA/MRSA screening (Methicillin-sensitive *S. aureus*) with nostrils and cutaneous (axillary and perineal) swabs, body and intraoral decolonization before surgery, and body and nasal eradication in the case of an MSSA- or MRSA-positive screening [14,15]. The focus of pre-operative care is the decolonization of cutaneous endogenous sources of infection.

In the intra-operative phase, we observe several cautions, like using chlorhexidine-alcohol-based prep, changing surgical gloves every 60′–90′ and before handling implants, using laminar airflow ventilation, warming devices to prevent hypothermia, using suction drains, performing pocket irrigation with antiseptic antibacterial 10% betadine double-antibiotic solution, and performing triple-antibiotic irrigation of implants in packaging. In terms of the type of antibiotic prophylaxis, commonly we use antibiotics including an intravenous first- or second-generation cephalosporin. This is administered 30–60 min before the surgical skin incision. In patients with allergies to β-lactam antibiotics, we use a non-β-lactam antibiotic with an adequate spectrum, such as clindamycin. In contrast, in patients who tested positive for MRSA, we use a combination of vancomycin and gentamicin. In the post-operative phase, we administer in high-risk patients prolonged antibiotic prophylaxis with cefalexin or clindamycin in case of a penicillin allergy. We remove drains when effluent totaled 30 mL or less for 2 consecutive days or by post-operative day 21 Box 1, Box 2 and Box 3.

Box 1 **Prevention Protocol for SSIs—Pre-operative phase.**

                                                                **PRE-OPERATIVE-PHASE**

Screening for MSSA/MRSA
(up to 6 weeks prior to surgery):

- Nostrilis swab
- Cutaneous (axillary and perineal) swab

Decolonization:

- Body washing with chlorhexidine 4% (daily, from 3 days before surgery)
- Intraoral washing with chlorhexidine oral rinse (on the day of surgery)

Eradication if tested positive for MSSA:

- Body washing with chlorhexidine 4% (daily, from 3 days before surgery)
- Muciprocin 2% nasal ointment (applied three times daily, from 3 days before surgery)

Eradication if tested positive for MRSA:

- Body washing with chlorhexidine 4% (daily, from 5 days before surgery)
- Muciprocin 2% nasal ointment (applied three times daily, from 5 days before surgery)
- Re-screening 48–72h after eradication protocol *

MSSA, Methicilin.sensitive *S. aureus;* MRSA, Methicilin-resistant *S. aureus;* SSIs, surgical site infections. * It is mandatory to have 3 negative screenings before surgery, done at a time frame of 7 days or more after the eradication protocol, which could be administered maximum twice; if the patient keeps being tested positive for MRSA, administer adequate intravenous antibiotic prophylaxis before surgery and if possible, isolate the patient.

Box 2 **Prevention Protocol for SSIs—Antibiotic prophylaxis.**

                                                            **ANTIBIOTIC PROPHYLAXIS**

Intravenous antibiotic prophylaxis at the time of induction, for every patient:

- Cefazolin 2g;
- OR Clindamycin 600 mg, if penicillin or cephalosporins allergies;
- Vancomycin 15 mg/kg + Gentamicin 3 mg/kg, if patient positive for MRSA

Inytavenous 24-h multiple dose antibiotic prophylaxis:

- Cefazolin 1 g q8hr;
- OR Clindamycin 600 mg q8hr, if penicillin or cephasporins allergies;

Prolomged post-operative antibiotic prophylaxis, in high-risk patients:

- Cefalexin 500 P.O. q6hr;
- OR Clindamycin 300 mg P.O. q8hr, if penicillin or cephalosporins allergies

P.O., oral administration.

Box 3 **Prevention Protocol for SSIs—Intra-operative phase.**

                                                              **INTRA-OPERATIVE-PHASE**

- Surgical hand preparation with antimicrobial soap and water or alcohol-based hand rub before donning sterile gloves
- Preparation of the skin prior to draping using 2% chlorhexidine with 70% isopropyl alcohol
- Perform careful atraumatic pocket dissection and careful hemostasis
- Change surgical gloves every 60′ to 90′ and before handling implants
- Perform pocket irrigation *
- Minimize implant open time to reduce contamination
- Use a “minimal or no-touch” technique where possible
- Use closed suction drains to reduce hematoma or seroma formation in selected cases, “tunneling” them into a subcutaneous plane
- Warming devices should be used to prevent hypothermia
- It is recommended to reduce the operating time
- Laminar airflow ventilation system

* There is a paucity of data supporting one form of washout over another. At our institution, we perform pocket and implant washing with antiseptic antibacterial 50% betadine double-antibiotic solution.

In group 2, good-practice rules were followed without a standardized protocol. MSSA/MRSA infective assessment was not performed, and prophylactic measures were not taken. Iodopovidone solution was used for disinfection, gloves were changed before handling implants, and pocket and implant irrigation was performed with NaCl 0.9% solution. Meticulous hemostasis was ensured, and drains were placed and removed as early as possible. Patients were not stratified by risk and were treated uniformly with amoxicillin/clavulanic acid for one week.

Pertinent data collected from the patient records were age at the time of surgery, type of reconstruction, BMI > 30, smoking status, previous radiotherapy, immunosuppression, post-operative complications and their management, comorbidities, radiotherapy, chemotherapy, and any subsequent surgery.

The data were collected and managed using Microsoft Excel (Microsoft Office 365). The statistical analysis was performed and completed for both the cohorts using the Mann–Whitney test and the χ^2^ test with the SPSS statistics 28 software. Predictive factors with *p* < 0.1 in the univariate analyses were included in the multivariate analysis. A two-tailed *p* < 0.05 was considered statistically significant.

We performed a logistic regression to control for clinically relevant differences between the cohorts by including in the statistical model only those variables found to be significant at univariate statistical analysis (*p*-Value < 0.05), including the type of surgery conducted (e.g., mastectomy) and risk factors (e.g., smoking) for the development of complications.

## 3. Results

In the study, 396 female patients were enrolled: 295 patients in group 1 (with the use of the prevention protocol in the hospital of Trieste) and 101 patients in group 2 (without the use of the prevention protocol). In group 2, two patients had two reconstructive attempts, and both failed, so they were considered twice in the count.

The mean age in group 1 was 52.61 ± 12.15, while in group 2 it was 54.03 ± 9.65 (*p* = 0.236).

There were 221 immediate reconstructions: 167 with tissue expander, 6 with tissue expander and ADM, 34 reconstructions with direct implant, and 14 prepectoral implants and ADM. The delayed reconstructions with implant after tissue expander were 127, implant changings were 12, and contralateral implant positionings were 36. Among the infected cases, there were no patients with prepectoral reconstruction with ADM.

In group 1, as part of the protocol, patients underwent cutaneous and nostril swab testing for *Staphylococcus* MRSA/MSSA before surgery. Among infected patients, only one patient tested positive and received decolonization.

Regarding the post-surgical measures, in group 1, only two patients who developed an infection had risk factors for it (one was BMI, and the other was previous radiotherapy), so they were treated with prolonged antimicrobial therapy until drainage removal.

Concerning the timing of infection, in group 1, there were three early infections (onset < 60 days from surgery) and two late infections (onset > 60 days from surgery), whereas in group 2, infection cases had a late development, except for one patient.

Clinical signs and symptoms of infection included fever, redness, purulent fluid discharge, swelling, and pain. When fluids could be collected, they were sent for microbiological analysis. A prevalence of bacterial infection (*S. aureus*, *S. epidermidis*, and *S. haemolyticus*) and fungal infection (*Candida parapsilosis* and *Candida albicans*) was recognized. Three patients had negative microbiological tests despite clinical infection, possibly due to ongoing antibiotic treatment (Table 1).

When infection was suspected, empirical antimicrobial therapy with a wide spectrum was initiated. In group 2, antibiotics included minocycline and rifampicin.

If symptoms worsened or if there was not a clinical improvement from oral or intravenous antibiotics, patients underwent surgery to remove implants or tissue expanders and to perform a deep debridement. All specimens were sent for microbiological analysis. In group 1, as an attempt to save the reconstruction, in four cases a washing with saline solution and vancomycin was positioned for 48 h. None of the patients treated with saline wash solution experienced failure of reconstruction. Then, only saline solution irrigation for an additional five days was continued. Eventually, four patients underwent further reconstructive operation with tissue expanders, while one patient refused additional surgery. In group 2, all patients rejected further reconstruction.

Moreover, among the complications, the infection rate was statistically significant (*p* = 0.006), with a rate of 1.7% in group 1 and 7.9% in group 2.

In group 1, two patients had risk factors for infections and have been treated with prolonged antibiotics after surgery.

The most frequent pathogens were *Staphylococcus epidermidis* (30%), in two cases with methicillin resistance and fungal infection (30%) with *Candida albicans* and *Candida parapsilosis*, which were discovered in patients of group 2.

Three patients had negative swab results, even if there was clinical evidence of infection. In group 1, there were three early infections and two late infections, whereas in group 2, infections occurred with a late timing, except for one case.

Therefore, four patients in group 1 agreed to undergo further reconstructive surgery after implant explantations.

Other complications experienced were necrosis of the nipple–areolar complex, seroma, and wound dehiscence (16.4%), but these were not statistically significant.

Assessing the role of risk factors between the two groups, no significant differences were found, except for “tobacco use”, which was more frequent in group 1 than in group 2 (20.0% vs. 5.9%, *p*-Value < 0.001).

Independent study of risk factors in the two groups after stratification of enrolled patients into “Infected” and “Not infected” revealed no statistically significant differences in either group.

Logistic regression demonstrates that when all other variables were held constant, none of the included variables emerged as an independent predictor of prosthetic infection (Table 2).

## 4. Discussion

In implant-based reconstruction, infection stands out as one of the most frequent and concerning complications, with reported incidence rates varying from 1% to 35% in the literature. Over the years, several definitions of infection have been provided by the experts. Wound infection is considered the invasion by microorganisms that elicit a local, spreading, and/or systemic response in the host [16,17]. According to The International Wound Infection Institute (IWII), infection may present in five stages (contamination, colonization, local infection, spreading infection, systemic infection), depending on the ability of the host to overcome pathogens. Different clinical presentations of infection are related on the quality of microbes, on local conditions, and on the host defensive competence. This concept expands upon the definition provided by the Centers for Disease Control and Prevention (CDC), which categorizes infections as superficial, deep, and organ/space infections. It emphasizes that risk factors are pivotal in preventive strategies, particularly in reconstructive surgery involving implant devices like tissue expanders or definitive implants, which are associated with a higher infection risk [18,19].

Furthermore, breast implant infections are classified as “early” and “late” using a clinical breakpoint of 60 days post-surgery [20,21].

Despite various classifications, all authors agree on the profound impact of infection in the reconstructive pathway of oncological patients in terms of the psychological aesthetics effect, delay adjuvant therapy, and survival [19].

In the literature, only a few attempts have been conducted to elaborate preventive rules in breast surgery [22].

In 2020, we elaborated a comprehensive infection prevention protocol encompassing pre-operative, intra-operative, and post-operative measures, with the aim of trying to save reconstructions and to reduce hospitalization and morbidity [13]. In addition, other preventive measures, such as the application of incisional negative-pressure devices, have been introduced to limit complications.

In developing our protocol, we reviewed international guidelines [23,24], systematic reviews, and studies with high levels of evidence [18,25,26,27,28,29,30].

The results were inspiring, especially in comparison with our past retrospective infection rate (11.76%), prompting us to design a multi-centric retrospective study to analyze the potentialities of the protocol.

We compared data collected at the Hospital of Trieste, where the protocol is employed daily, and at the Hospital of Gorizia, where members of the same surgery team take different prevention cautions.

A total of 396 reconstructions were performed, with 295 in group 1 and 101 in group 2. Infected patients were five in the first group (1.7%) and eight in the second one (7.9%), with a global infection rate of 3.2%.

In the study, most infections (70%) occurred after 60 days after surgery.

These findings indicate a decrease in infection rates following protocol implementation, potentially attributed to its positive effects. We believe this reduction in infections may be linked to appropriate surgical indications and the measures taken for patients with risk factors.

Indeed, we analyzed all the risk factors that could have influenced the infection rate in both groups. There was not statistically a difference in the distribution of risk factors among patients of both groups except for “tobacco use”, which was more recurrent in group 1 than in group 2 (20.0% vs. 5.9%, *p*-Value < 0.001).

Interestingly, despite having more smoking patients in group 1, the overall rate of post-operative infection was lower. Notably, no smoking patients in group 1 developed a prosthetic infection during follow-up, whereas two patients in group 2 who developed a post-operative prosthetic infection were smokers.

Given that tobacco use is known to increase the risk of prosthetics infection, although all other risk factors were equal, we can reasonably argue that the protocol we implemented serves as a protective factor against post-operative infectious risks, especially in female smokers.

Regarding pathogens, the most common were *Staphylococcus epidermidis* (30%), in two cases with methicillin resistance and fungal infection (30%) with *Candida albicans* and *Candida parapsilosis*.

Thanks to the multidisciplinary collaboration with the staff of The Infectious Diseases Unit, patients received empirical broad-spectrum antibiotics. Following a microbiological definitive response, therapy was tailored based on antibiogram results and included dalbavancin, rifampicin, and antifungal drugs, after excluding any allergies or intolerances [31,32].

In cases of worsening local conditions, surgical removal of tissue expanders or implants was planned, accompanied by deep debridement and the application of saline solution with vancomycin for 48 h, followed by saline solution alone for an additional 5 days. These measures allowed secondary surgical reconstruction, delayed about 15 days after primary implants removal. No patient treated with saline wash solution experienced failure of reconstruction.

However, there are still risk issues that require examination to understand their impacts and effects on breast reconstruction, for example, the presence of biofilm on the implant’s surfaces.

Suspected biofilm formation arises when appropriate antibiotic treatment fails, accompanied by low-level local symptoms and delayed healing despite optimal wound management. This underlines the necessity of a radical debridement of the forming capsule when suspecting an infection with biofilm, because according to its nature, it is barely achievable by empirical antibiotics [33].

Moreover, the timing of wound swabs and aspirates should be considered. In fact, for the patients with negative microbiological responses, antimicrobial therapy was already started, and there was not a standard duration according to multidisciplinary consultation, so a distortion of the result may have occurred.

Anyway, there are still procedures that could be standardized, even if there is a lack of common consensus in the literature. One example is the management of dressings, especially for drainages, but also for wound sites. In our practice, occlusive dressings were made with sterile gauzes sliced in the middle to embrace the tube, and they were changed every 48 h with a sterile technique. In addition, in case of doubt regarding the vitality of mastectomy flaps, incisional negative-pressure medications are applied to reduce the risk of seroma and wound dehiscence [34]. In future studies, there could be an improvement of the preventive protocol also in considering dressings and their timing of changing.

Furthermore, another topic deserving detailed study is the approach to patients with a definitive diagnosis of infection. Some authors argue on the best surgical approach to avoid complications using implants or autologous techniques, while others argue on the possibility of saving implants with antibiotics or proceeding to explantations [35,36,37,38,39,40]. Bearing in mind all the viable options, we employed a multistep method, with a discussion with the experts in the field of infectious disease and the microbiology laboratory [41].

Several limitations exist in our study. Firstly, its retrospective nature precludes extending the results to future perspectives or different care settings. Secondly, while the clinical characteristics of the patient sample are representative, factors such as the comorbidity burden and frailty status were not objectively assessed due to the study’s retrospective nature. Lastly, although a power analysis was not conducted due to the retrospective observational design, the sample size remains a significant limitation, given the low baseline rates of breast prosthetic infections. Future studies with larger cohorts are needed to validate our findings. We aspire to refine our study and establish standards for the comprehensive treatment of infected patients undergoing secondary reconstruction attempts in the future.

## 5. Conclusions

Implant-based breast reconstruction is included in the most employed reconstruction techniques after mastectomy. SSIs are clearly a significant surgical complication in breast reconstruction with implants, often leading to a longer hospital stay, increased healthcare costs, and the potential loss of reconstruction, which can profoundly impact patients. Moreover, since the introduction of the prevention protocol for SSIs in June 2020, the overall infection rate has been 1.7% among patients undergoing breast reconstruction with implants. Despite the study limitations, we believe that establishing a standardized pathway encompassing pre-, intra-, and post-operative measures is crucial for providing effective and consistent treatment for patients. Additionally, it serves as a foundation for conducting more comprehensive analyses of complications and outcomes in breast reconstruction with implants.

Following the introduction of our prevention protocol, we initiated a study to assess the influence of risk factors on infection rates. We observed a decreased incidence of infection following breast surgery involving implants or tissue expanders.

## Figures and Tables

**Table 1:** Patients who developed infections in group 1 (experimental group) and in group 2 (control group), demographical characteristics (age, type of reconstructions), risk factors, pathogens, timing of onset of infections, and need for further procedures.

Group	Age	Side	Mast.	Reconstruction	Immunodepression	DMII	BMI	Tobacco Use	Previous RT	Pathogen	Timing	Failure of Reconstruction
1	63	L	No	Tissue expander to implant	No	No	<30	No	No	*S. aureus totisensibile*	Late	Yes
1	58	L	Yes	Tissue expander	No	No	≥30	No	No	Not found	Early	No
1	73	L	Yes	Tissue expander	No	No	<30	No	No	*S.MRS Epidermidis*	Early	No
1	45	L	No	Tissue expander to implant	No	No	<30	No	No	*S.MRS Epidermidis*	Early	No
1	45	L	Yes	Tissue expander	No	No	<30	No	Yes	Not found	Late	No
2	66	R	Yes	Tissue expander	No	No	≥30	No	No	Not found	Late	Yes
2	47	R	Yes	Tissue expander	No	No	<30	No	No	*Candida parapsilosis*	Late	Yes
2	47	L	Yes	Tissue expander	No	No	<30	No	No	*Candida parapsilosis*	Late	Yes
2	66	L	Yes	Tissue expander	No	No	<30	No	Yes	*S. haemolitycus*	Late	Yes
2	63	R	Yes	Tissue expander	No	No	<30	No	No	*S. epidermidis*	Early	Yes
2	56	L	Yes	Tissue expander	Yes	No	≥30	No	No	*S. epidermidis*	Late	Yes
2	64	L	Yes	Tissue expander	No	No	<30	Yes	No	*Candida albicans*	Late	Yes
2	64	L	Yes	Implant	No	No	<30	Yes	No	*Candida albicans*	Late	Yes

R: right, L: left; Early = onset < 60 days from surgery, Late = onset > 60 days from surgery.

**Table 2:** Association between risk factors and infection.

Risk Factors	Group 1	Group 2	*p*-Value
Immunodepression (%)	23 (7.8)	12 (11.9)	0.212
DM II (%)	13 (4.4)	3 (3.0)	0.770
BMI > 30	19 (6.4)	7 (6.9)	0.864
Tobacco use	59 (20.0)	6 (5.9)	<0.001
Previous RT (%)	32 (10.8)	17 (16.8)	0.115
Group 1			
**Risk factors**	**Infected** (n = 5)	Not infected (n = 290)	*p*-Value
Immunodepression (%)	0 (0.0%)	23 (7.9%)	1.000
DM II (%)	0 (0.0%)	13 (4.5%)	1.000
BMI > 30 (%)	1 (20.0%)	18 (6.2%)	0.285
Tobacco use (%)	0 (0.0%)	59 (20.3%)	0.587
Previous RT (%)	1 (20.0%)	31 (10.7%)	0.439
Group 2			
**Risk factors**	**Infected** (n = 8)	Not infected (n = 93)	*p*-Value
Immunodepression (%)	1 (12.5%)	11 (11.8%)	1.000
DM II (%)	0 (0.0%)	3 (3.2%)	1.000
BMI > 30 (%)	2 (25.0%)	5 (5.4%)	0.095
Tobacco use (%)	2 (25.0%)	4(4.3%)	0.070
Previous RT (%)	1 (12.5%)	16 (17.2%)	1.000

Groups were comparable in terms of type of surgical procedures and risk factors.

## Data Availability

The data presented in this study are available in the article.
